# Omega-3 PUFAs in musculoskeletal health and sports medicine: from molecular pathways to precision nutrition strategies

**DOI:** 10.3389/fnut.2026.1789924

**Published:** 2026-04-02

**Authors:** Hao Zhang, Minshun Zhu, Yuhao Qu, Xuebo Zhang, Zhimin Wu, Zheng Wang

**Affiliations:** 1 Department of Orthopedics, Sheng'zhou Hospital of Traditional Chinese Medicine, Sheng'zhou, China; 2 Department of Rehabilitation, Lu'an Hospital of Traditional Chinese Medicine, Lu'an, China; 3 Department of Emergency, Yan'tai Hospital of Traditional Chinese Medicine, Yan'tai, China; 4 Department of Orthopedics, Wen'deng Orthopedic Hospital, Wen'deng, China; 5 Department of Orthopedics, Shao'xing Hospital of Traditional Chinese Medicine, Shao'xing, China; 6 Department of Orthopedics, The First Affiliated Hospital of Anhui University of Chinese Medicine, He'fei, China

**Keywords:** bone-muscle axis, chronic low-grade inflammation, inflammation, musculoskeletal health, precision nutrition, resolution, ω-3 PUFAs

## Abstract

The imbalance in the dietary ω-6/ω-3 polyunsaturated fatty acid (PUFA) ratio contributes to chronic low-grade inflammation, a key pathological basis for degenerative musculoskeletal conditions such as osteoporosis and osteoarthritis. While individual reviews have explored aspects of ω-3 PUFAs in bone or muscle, a comprehensive narrative review integrating their multidimensional mechanisms with translational evidence across orthopedics and sports medicine is less common. This review consolidates evidence from fundamental science, clinical trials, and translational research, employing an interdisciplinary approach to systematically elucidate their mechanisms and application values. ω-3 PUFAs exert multi-layered anti-inflammatory and pro-repair effects through mechanisms including cell membrane remodeling, reprogramming of lipid mediator profiles (promoting the generation of specialized pro-resolving mediators), and inhibition of key pathways such as NF-κB and the NLRP3 inflammasome. Clinical studies indicate that ω-3 PUFA supplementation can improve bone metabolic markers, alleviate pain in osteoarthritis, and reduce inflammation and muscle loss in the perioperative period. In sports medicine, ω-3 PUFAs enhance muscle anabolic metabolism, optimize energy utilization, promote recovery, and demonstrate neuroprotective potential. Their extended application value lies in regulating the “bone-muscle axis,” providing targeted nutritional support for athletes, and serving as a nutritional countermeasure in extreme environment medicine. Statement of Significance: This review constructs an integrative framework linking molecular mechanisms to clinical translation, positioning ω-3 PUFAs as key physiological modulators for musculoskeletal health and delineating future directions for precision nutrition-based intervention strategies.

## 1 Introduction

The health of the skeletal muscle system is crucial for maintaining human structural integrity and function, with its homeostasis regulated by various factors including mechanical forces, metabolism, aging, and injury (1, 2). Traditional nutritional interventions have primarily focused on nutrients such as calcium and vitamin D. Recent research, however, has identified ω-3 polyunsaturated fatty acids (PUFAs), mainly eicosapentaenoic acid (EPA) and docosahexaenoic acid (DHA), as important regulators of skeletal muscle homeostasis (3, 4).

Modern diets, characterized by a high ω-6/ω-3 ratio, drive a pro-inflammatory state (5–9), which is a key pathological driver of degenerative conditions such as osteoporosis, sarcopenia, and osteoarthritis, and may also impair post-exercise tissue repair (10). Consequently, modulating ω-3 PUFA levels through nutritional strategies has emerged as a research direction for the prevention and management of related diseases and for supporting exercise rehabilitation (11).

Mechanistically, ω-3 PUFAs act through multiple pathways. Their unique molecular structure alters cell membrane fluidity, influencing membrane protein function and mechanosensitive channel activity (12, 13). They serve as precursors for specialized pro-resolving mediators (SPMs), facilitating the orderly resolution of inflammation. By competitively inhibiting the synthesis of pro-inflammatory eicosanoids and downregulating pathways such as NF-κB and the NLRP3 inflammasome, they help shape an anti-inflammatory microenvironment (14–16). Furthermore, ω-3 PUFAs regulate key cellular processes including osteogenic differentiation, bone resorption, muscle protein synthesis, and cartilage metabolism (17–20).

While individual reviews have explored aspects of ω-3 PUFAs in bone or muscle, a comprehensive synthesis that integrates their multidimensional mechanisms with translational evidence across orthopedics and sports medicine is less common. This article aims to fill this gap by providing an interdisciplinary framework, connecting molecular pathways to clinical applications, with a particular focus on emerging paradigms such as precision nutrition and the bone-muscle axis, to provide a reference for future interventions.

### 1.1 Approach to literature selection

Given the broad and interdisciplinary scope of this review, a comprehensive but non-systematic literature search was conducted to identify relevant studies. The search was performed in the electronic databases PubMed, Web of Science, and Scopus, covering the period from January 2000 to December 2024. Key search terms included combinations of: “omega-3 polyunsaturated fatty acids,” “eicosapentaenoic acid,” “docosahexaenoic acid,” “bone,” “muscle,” “skeletal muscle,” “osteoporosis,” “osteoarthritis,” “fracture healing,” “exercise,” “sports performance,” and “inflammation.” The reference lists of retrieved articles were also manually screened to identify additional relevant publications. Priority was given to peer-reviewed original research articles, systematic reviews, meta-analyses, and clinical guidelines. This is a narrative review; therefore, a formal systematic review protocol with pre-specified inclusion/exclusion criteria and risk of bias assessment was not applied. The selection of literature was guided by the authors’ expertise to provide a balanced and comprehensive overview of the field, with an emphasis on studies that elucidate molecular mechanisms, report clinical outcomes, or explore translational applications.

## 2 Dietary sources and physiological significance of ω-3 PUFAs

### 2.1 Major dietary sources and intake recommendations

Dietary sources of ω-3 polyunsaturated fatty acids (PUFAs) are diverse. To maintain health, the World Health Organization recommends a daily intake of 0.25–2 grams of EPA + DHA for adults (21). Key practical approaches include consuming 2–3 servings (approximately 100–150 grams per serving) of oily fish per week (22); using vegetable oils rich in alpha-linolenic acid (ALA), such as canola oil; and moderately consuming walnuts and flaxseeds to increase ALA intake (23). For individuals unable to meet requirements through diet alone, high-quality fish oil or algal oil supplements represent a viable alternative (24). Table 1 summarizes the dietary sources, forms, and recommendations of ω-3 PUFAs.

**Table 1:** Dietary sources, forms, and recommendations of omega-3 polyunsaturated fatty acids (PUFAs).

Source category	Typical foods	Main forms and characteristics	Recommendations	References
Marine animals	Salmon, mackerel, sardines, herring, trout; oysters, mussels	Directly provide EPA and DHA with the highest bioavailability.	Consume 2–3 servings (each ~100–150 g) of fatty fish per week. Prefer cooking methods such as baking and steaming.	(22)
Terrestrial plants	Flaxseeds, chia seeds, walnuts, canola oil, soybeans	Rich in ALA, suitable for vegetarians, but the conversion to EPA and DHA is limited.	Daily intake of ground flaxseeds or chia seeds (about 1 tablespoon), a small handful of walnuts, and cooking with canola oil are recommended.	(23)
Fortified foods and supplements	ω-3 fortified milk, eggs; fish oil, algae oil supplements	Convenient to use, applicable for populations with insufficient intake or specific needs.	Pay attention to purity, oxidative stability, and dosage when selecting supplements, and consult professional medical advice.	(23)

This table succinctly summarizes the three primary dietary sources of ω-3 polyunsaturated fatty acids (PUFAs): marine animals provide EPA and DHA with high bioavailability; terrestrial plants are rich in ALA but have limited conversion efficiency; and fortified foods and supplements offer convenient intake pathways for specific populations, accompanied by specific intake recommendations.

### 2.2 Physiological and public health significance of adequate intake

Adequate intake of ω-3 PUFAs holds dual core significance: it serves as the material foundation for their physiological functions and represents an essential public health strategy for correcting dietary imbalances and countering chronic inflammation (25).

From a physiological perspective, the synthesis of long-chain ω-3 PUFAs (EPA and DHA) with direct bioactivity is highly inefficient in humans. The conversion of plant-derived ALA is limited (typically <10%) and is readily competitively inhibited by a high dietary ratio of ω-6 fatty acids (26). Therefore, direct dietary intake of EPA and DHA is the most reliable way to maintain effective concentrations in target tissues such as bone and muscle. The erythrocyte membrane ω-3 index, which reflects long-term nutritional status, has been associated with lower systemic inflammation levels and enhanced muscle protein anabolic responses (19, 27).

**Figure 1:**
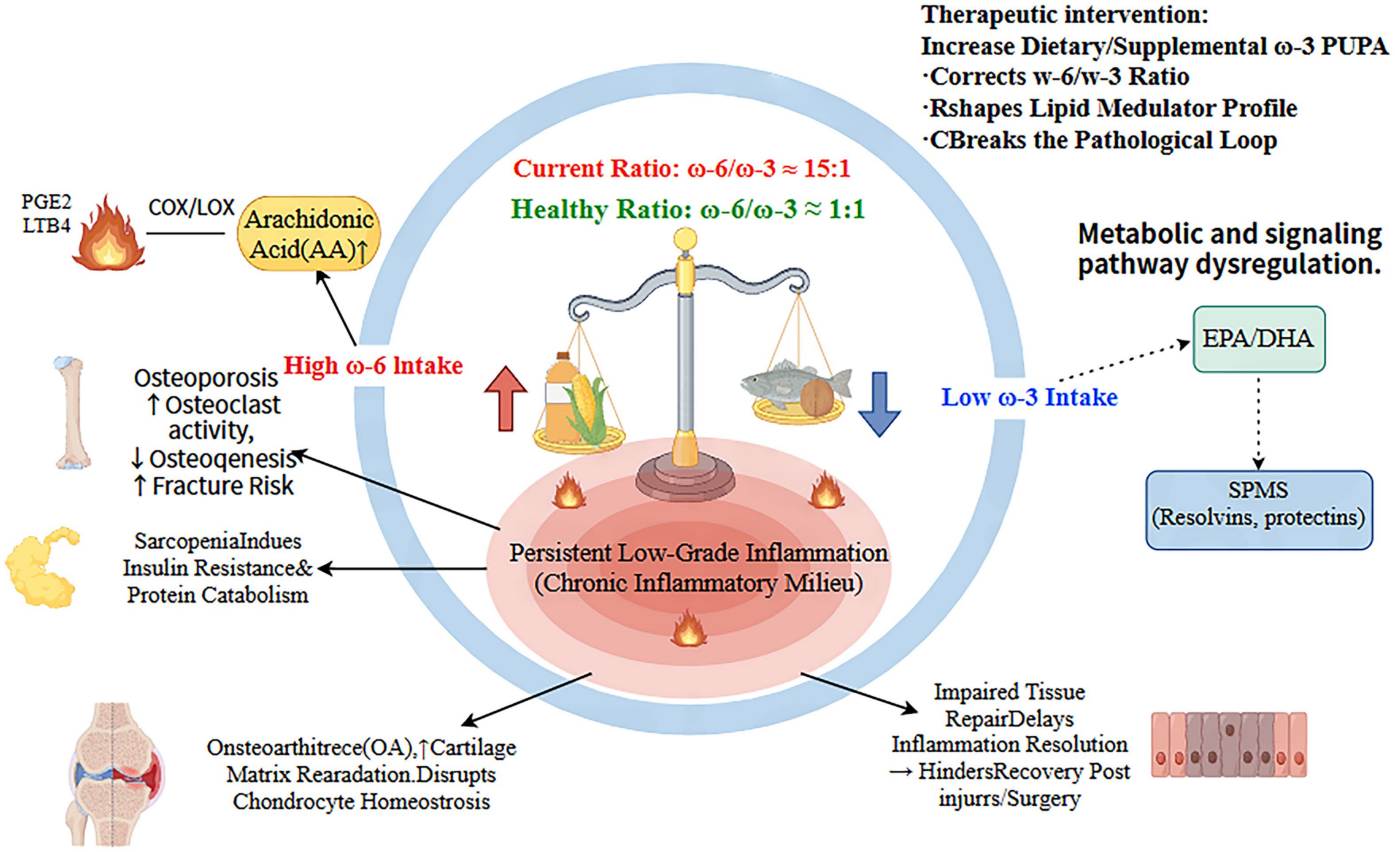
Pathophysiological loop linking dietary ω-6/ω-3 imbalance to chronic low-grade inflammation and musculoskeletal degeneration. The prevalent modern diet, characterized by a high intake of ω-6 PUFAs from vegetable oils and a low intake of ω-3 PUFAs, leads to a significantly elevated ω-6/ω-3 ratio (~15:1), deviating from the evolutionary-adapted ratio (~1:1). This imbalance causes a metabolic and signaling shift: an overabundance of arachidonic acid (AA) fuels the excessive production of potent pro-inflammatory eicosanoids (e.g., PGE_2_, LTB_4_), while a relative deficiency of EPA/DHA limits the synthesis of specialized pro-resolving mediators (SPMs). The net result is a state of persistent low-grade inflammation. This chronic inflammatory milieu serves as a common pathological foundation that drives multiple degenerative processes in the musculoskeletal system, including osteoporosis (via enhanced bone resorption), sarcopenia (via induced anabolic resistance), osteoarthritis (via cartilage matrix destruction), and impaired repair after injury/surgery. Increasing ω-3 PUFA intake (dietary or supplemental) is a foundational nutritional strategy to correct this imbalance and break the vicious cycle. LTB_4_, leukotriene B4. (This figure was created using www.figdraw.com. ID: YPIUU73e35).

Contemporary industrialized dietary patterns have led to a severe imbalance in ω-6/ω-3 intake ratios (often as high as 15:1), significantly deviating from the evolutionary-adapted ratio of approximately 1:1 (5). This imbalance has defined pathophysiological consequences: excess ω-6 fatty acids (primarily arachidonic acid) metabolically dominate, promoting the generation of potent pro-inflammatory mediators such as prostaglandin E₂ (PGE₂) and leukotriene B₄ (LTB₄), while relatively suppressing the production of pro-resolving mediators (e.g., resolvins) derived from ω-3 PUFAs (4). The resulting state of chronic low-grade inflammation constitutes a key pathological basis for diseases like osteoporosis, sarcopenia, and osteoarthritis (7–9). The relationship between ω-6/ω-3 imbalance and chronic low-grade inflammation as well as musculoskeletal degeneration is illustrated in Figure 1.

Thus, increasing ω-3 PUFA intake is a fundamental intervention aimed at correcting nutritional imbalances at their source, reshaping metabolic patterns, and constructing a more favorable internal environment for skeletal muscle health.

## 3 Fundamental scientific mechanisms of ω-3 PUFAs

### 3.1 Physicochemical basis of molecular structure

The biological activity of ω-3 PUFAs stems from their unique molecular structure, characterized by the first cis double bond located between the third and fourth carbon atoms. The multiple cis double bonds in eicosapentaenoic acid (EPA) and docosahexaenoic acid (DHA) create a bent molecular conformation, which serves as the core starting point for their distinctive biophysical and biochemical effects (28).

### 3.2 Core molecular mechanisms

#### 3.2.1 Cell membrane remodeling and mechanobiology

ω-3 PUFAs, particularly DHA, incorporate into the phospholipid bilayer of cell membranes. By increasing membrane fluidity and altering its mechanical properties, they directly influence membrane protein function (29). For instance, DHA can specifically enhance the membrane tension sensitivity of the mechanosensitive ion channel Piezo1 (30). This channel is a key sensor for mechanical stimuli such as fluid shear stress and stretch in osteoblasts, osteocytes, and muscle cells (31), thereby laying the initial mechanical foundation for subsequent adaptive growth and repair in the skeletal muscle system.

#### 3.2.2 Reprogramming of lipid mediator profiles: shifting from pro-inflammatory to pro-resolving

In the realm of inflammation and immune regulation, ω-3 PUFAs play a fundamental role by competitively reshaping the spectrum of lipid mediator production. EPA shares metabolic pathways (e.g., cyclooxygenase/COX and lipoxygenase/LOX) with the major pro-inflammatory precursor, arachidonic acid (AA). When dietary EPA levels are sufficient, it competitively displaces AA as the substrate for these enzymes. This leads to a critical shift: reduced generation of potent series-2 prostaglandins (e.g., PGE₂) and thromboxanes from AA, replaced by series-3 metabolites from EPA (e.g., PGE₃) with significantly attenuated bioactivity (32). This transformation represents the first step in reducing “pro-inflammatory signals” at their source.

More importantly, ω-3 PUFAs are not only about “reducing bad signals” but also serve as precursors for generating “good signals.” They can be further metabolized into a class of active molecules termed specialized pro-resolving mediators (SPMs), including resolvins, protectins, and maresins. Unlike traditional anti-inflammatory drugs, SPMs do not simply suppress inflammation; instead, they actively emit programmed “stop” and “repair” signals. They inhibit further neutrophil recruitment, promote macrophage switching to a repair phenotype, and enhance efferocytosis (clearance of apoptotic cells), thereby actively guiding the orderly resolution of inflammation and initiating tissue repair (33). While the role of SPMs in actively resolving inflammation is well-established in pre-clinical models (34, 35), direct evidence for their causal role in mediating the clinical benefits of ω-3 supplementation in humans is still emerging and represents an active area of investigation.

#### 3.2.3 Direct inhibition of key intracellular pro-inflammatory pathways

Beyond modulating lipid mediators, ω-3 PUFAs and their metabolites can directly act upon core intracellular pro-inflammatory signaling pathways. On one hand, they can interfere with and inhibit the activation of nuclear factor kappa B (NF-κB), a master switch, by activating cell membrane receptors such as GPR120, thereby reducing the gene transcription of key pro-inflammatory cytokines like tumor necrosis factor-alpha (TNF-*α*) and interleukin-6 (IL-6) (15). On the other hand, DHA and EPA have been shown to effectively inhibit the assembly and activation of the NLRP3 inflammasome (16). The NLRP3 inflammasome is a core intracellular platform that senses damage or danger signals and catalyzes the production of the potent inflammatory cytokine IL-1β, playing a key role in inflammatory bone loss and joint destruction (36). ω-3 PUFAs may prevent the overactivation of this platform through mechanisms such as stabilizing mitochondrial function and reducing reactive oxygen species generation, thereby curbing the release of destructive factors like IL-1β.

### 3.3 Targeted modulation at the cellular functional level

#### 3.3.1 Effects on osteoblasts: promoting bone formation

In osteoblasts, ω-3 PUFAs (particularly DHA) can activate the canonical Wnt/β-catenin signaling pathway. By stabilizing β-catenin protein and promoting its nuclear translocation, they enhance the activity of key osteoblast transcription factors such as Runx2, driving osteoblast differentiation and functional maturation (17).

#### 3.3.2 Effects on osteoclasts: inhibiting bone resorption

Osteoclast activity is strictly regulated by the RANKL/RANK/OPG system. ω-3 PUFAs (with more studies on EPA) intervene in this system through multiple approaches: reducing the production of the pro-osteoclastogenic signal RANKL, increasing the secretion of its inhibitory factor OPG, thereby altering the local RANKL/OPG balance; simultaneously, they can directly interfere with RANKL-induced downstream signaling in osteoclast precursors, inhibiting their differentiation into mature osteoclasts (18). The net result is effective suppression of excessive bone resorptive activity.

#### 3.3.3 Effects on skeletal muscle cells: optimizing anabolic metabolism and homeostasis maintenance

In skeletal muscle, a core function of ω-3 PUFAs is to enhance cellular sensitivity to anabolic signals. Studies show that supplementation with EPA and DHA increases the responsiveness of muscle cells to insulin and amino acids. The primary mechanism involves enhancing the sensitivity of the PI3K/Akt signaling pathway upstream of mTORC1. This ‘sensitizing’ effect means that in the presence of ω-3 PUFAs, a given dose of insulin and amino acids can more effectively activate mTORC1 and its downstream protein synthesis machinery (19). It is important to note that ω-3 PUFAs do not appear to directly activate mTORC1 independently. Additionally, ω-3 PUFAs (especially DHA) have been found to regulate muscle autophagy, aiding in the clearance of damaged organelles, which is crucial for maintaining muscle quality control and metabolic health under stress (37).

#### 3.3.4 Effects on chondrocytes: maintaining articular cartilage homeostasis

In articular cartilage, ω-3 PUFAs exert protective effects through various mechanisms. They downregulate the expression of matrix metalloproteinases (MMPs), which are responsible for degrading the cartilage matrix and are induced by pro-inflammatory factors. Concurrently, they upregulate the levels of their natural inhibitors (TIMPs), thereby remodeling the MMP/TIMP balance and slowing pathological matrix destruction (20). Combined with their anti-inflammatory and SPM-generating capabilities, ω-3 PUFAs create a more favorable environment for chondrocyte survival and synthetic function, helping to delay the pathological progression of osteoarthritis.

**Figure 2:**
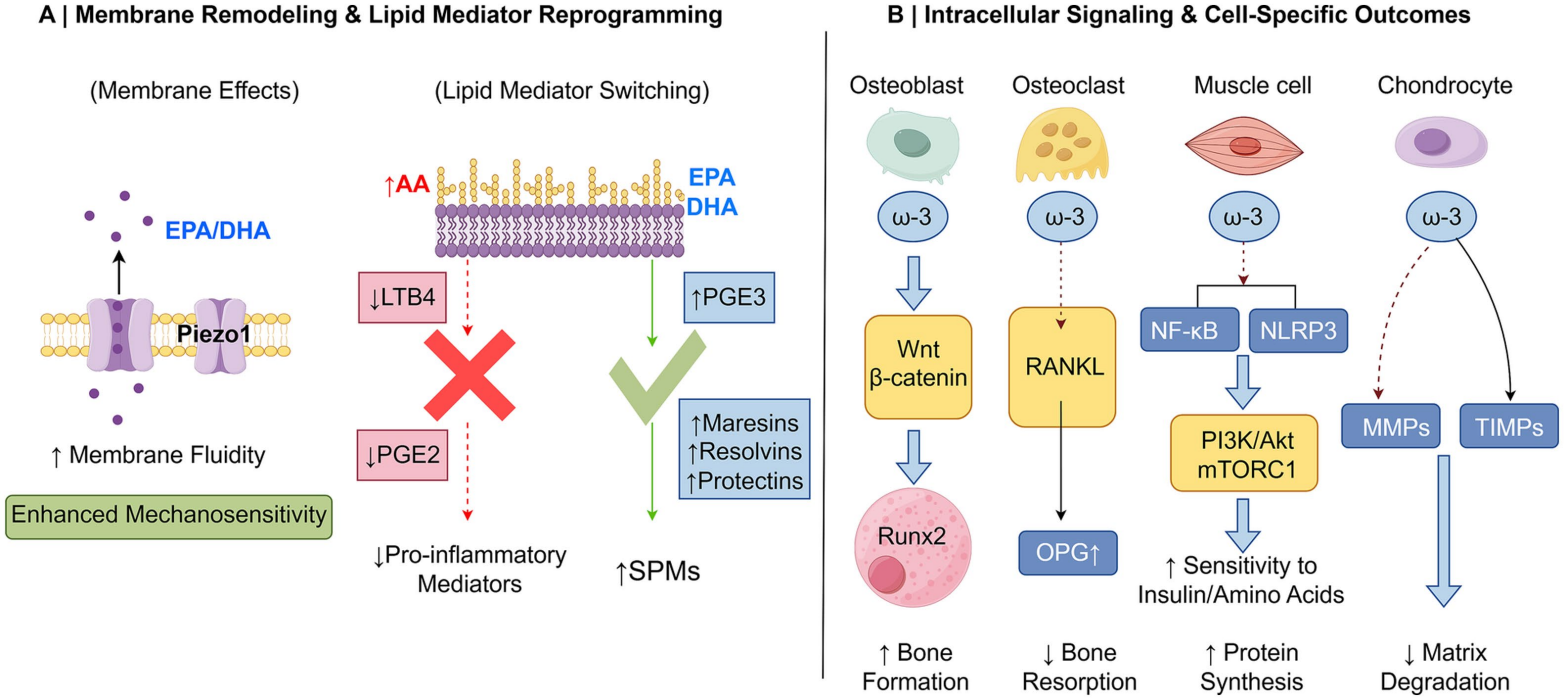
Schematic illustration of core mechanisms by which ω-3 PUFAs regulate skeletal muscle health. This figure depicts the multi-layered mechanisms of action of omega-3 polyunsaturated fatty acids (ω-3 PUFAs), specifically eicosapentaenoic acid (EPA) and docosahexaenoic acid (DHA), in regulating the health of the musculoskeletal system. To enhance clarity, the complex pathways are divided into two complementary panels. **(A)** Membrane remodeling and lipid mediator reprogramming: This panel illustrates the proximal events at the cell membrane level. Left side: ω-3 PUFAs (EPA/DHA) are incorporated into the phospholipid bilayer, increasing membrane fluidity and enhancing the sensitivity of mechanosensitive ion channels like Piezo1, which primes cells for mechanical signal transduction. Right side: ω-3 PUFAs competitively inhibit the metabolism of arachidonic acid (AA, an ω-6 PUFA), leading to reduced production of potent pro-inflammatory mediators such as prostaglandin E_2_ (PGE_2_) and leukotriene B_4_ (LTB_4_). Concurrently, EPA and DHA serve as precursors for the synthesis of specialized pro-resolving mediators (SPMs), including resolvins, protectins, and maresins, which actively promote the resolution of inflammation and tissue repair. The production of less bioactive series-3 prostaglandins (e.g., PGE_3_) from EPA also contributes to this shift from a pro-inflammatory to a pro-resolving lipid mediator profile. **(B)** Intracellular signaling and cell-specific functional outcomes: This panel details the downstream intracellular pathways activated by ω-3 PUFAs and their consequent functional effects in the four major cell types of the musculoskeletal system. Osteoblasts: ω-3 PUFAs (particularly DHA) activate the canonical Wnt/β-catenin signaling pathway, stabilizing β-catenin and promoting its nuclear translocation. This enhances the activity of the key transcription factor Runx2, driving osteoblast differentiation and bone formation. Osteoclasts: ω-3 PUFAs (particularly EPA) inhibit osteoclastogenesis by modulating the RANKL/RANK/OPG axis. They reduce RANKL production and increase osteoprotegerin (OPG) levels, thereby suppressing RANKL-induced signaling and decreasing bone resorption. Myocytes: ω-3 PUFAs enhance the sensitivity of muscle cells to anabolic stimuli (insulin and amino acids) by potentiating PI3K/Akt signaling, which leads to more effective activation of the mTORC1 pathway and a subsequent increase in muscle protein synthesis. Additionally, they inhibit pro-inflammatory pathways, including NF-κB and the NLRP3 inflammasome, reducing catabolic signals. Chondrocytes: ω-3 PUFAs help maintain cartilage homeostasis by downregulating the expression of matrix metalloproteinases (MMPs) and upregulating their endogenous inhibitors (TIMPs), thus shifting the MMP/TIMP balance towards matrix preservation and slowing cartilage degradation. Together, these two panels provide a comprehensive overview of how ω-3 PUFAs act from the cell membrane to the nucleus to exert their anti-inflammatory, pro-repair, and anabolic effects, thereby supporting overall skeletal muscle health. AA, arachidonic acid; PGE_2_, prostaglandin E_2_; LTB_4_, leukotriene B_4_; PGE_3_, prostaglandin E_3_; SPMs, specialized pro-resolving mediators; NF-κB, nuclear factor kappa B; NLRP3, NLR family pyrin domain containing 3; mTORC1, mammalian target of rapamycin complex 1; MMPs, matrix metalloproteinases; TIMPs, tissue inhibitors of metalloproteinases; OPG, osteoprotegerin; RANKL, receptor activator of nuclear factor-κB ligand. (This figure was created using www.figdraw.com. ID: ITITA44c8a).

In summary, ω-3 PUFAs do not act through isolated single targets. Instead, they establish a multi-dimensional, hierarchical system of action encompassing the modification of cell membrane physical properties, systemic reprogramming of lipid mediator and inflammatory signaling networks, and precise regulation of the fate and function of different cell types. These closely interrelated fundamental scientific mechanisms collectively form the theoretical foundation for understanding their clinical potential in the prevention and management of orthopedic diseases and the promotion of exercise health. The core mechanisms by which ω-3 polyunsaturated fatty acids regulate skeletal muscle health are shown in Figure 2.

## 4 Application of ω-3 PUFAs in the prevention and management of orthopedic diseases

The dosages of EPA + DHA used in clinical studies vary by context. For general bone health and osteoarthritis, doses in the range of 1–2 g/day are common. For enhancing muscle anabolic sensitivity in older adults or attenuating muscle loss in catabolic conditions (e.g., bed rest), higher doses of 2–4 g/day have been employed. In the sports medicine literature, doses for managing exercise-induced inflammation and muscle soreness typically range from >2 g/day, often initiated before the exercise bout. A summary of key clinical trials with specific dosages and outcomes is provided in Table 2.

**Table 2:** Summary of key clinical trials of ω-3 PUFAs in orthopedics and sports medicine.

Population	Intervention (Dose/form)	Duration	Comparator	Main outcomes related to musculoskeletal health	Key limitations
Healthy middle-aged/older adults (19)	3.4 g/d EPA + DHA (fish oil)	8 weeks	Olive oil	↑ Muscle protein synthesis response to insulin/amino acids	Small sample size; short duration
Postmenopausal women (39)	Variable EPA/DHA doses	Variable (3–24 mo)	Placebo or olive oil	↓ Bone resorption marker (CTX-1); variable effects on BMD	Heterogeneous studies in meta-analysis
Patients with knee OA (9)	>1 g/d EPA + DHA	3–6 months	Placebo or sunflower oil	↓ Pain (WOMAC, VAS); ↓ cartilage degradation biomarkers (CTX-II)	Mostly short-term; structural data limited
Older adults with sarcopenia (44)	ω-3 + leucine + vitamin D	13 weeks	Isocaloric control	↑ Muscle mass and function (gait speed)	Cannot isolate ω-3 specific effect
Healthy young adults (56)	>2 g/d EPA + DHA	Variable (pre/post exercise)	Placebo or olive oil	↓ Post-exercise muscle soreness (DOMS); ↓ markers of muscle damage (CK)	Variable exercise protocols; small samples
Patients post-hip arthroplasty (49)	ω-3 enriched immunonutrition	Perioperative (7–14 d)	Standard nutrition	↓ Post-operative infections; ↓ length of hospital stay	Part of a multi-nutrient formula
Bed rest (simulated spaceflight) (43)	2–4 g/d EPA + DHA	60 days	Standard diet	↓ Loss of bone mineral density (tibia); ↓ muscle atrophy	Highly controlled setting; small n
Healthy young men (59)	3 g/d fish oil	4 weeks (pre & post)	Safflower oil	↓ Perceived pain; ↓ inflammatory markers after eccentric exercise	Single bout of exercise; lab-based

This table summarizes representative clinical studies discussed in the paper, aiming to enhance the transparency of study selection and facilitate readers’ critical evaluation of the existing evidence. The table specifies each study’s population, intervention (dose/form), duration, comparator, main outcomes, and key limitations, covering multiple application areas such as osteoporosis, osteoarthritis, sarcopenia, and sports recovery.

### 4.1 Regulation of osteoporosis and fracture risk

Substantial research evidence supports the role of ω-3 PUFAs in bone health. In animal models simulating the postmenopausal state, such as ovariectomized (OVX) rats, supplementation with fish oil (providing EPA and DHA) demonstrates clear bone-protective effects. These studies show not only increased bone mineral density (BMD) but, more importantly, improvements in bone microstructure (e.g., trabecular number, thickness), translating directly into enhanced biomechanical performance (e.g., maximum load, stiffness) (38). The underlying mechanisms are closely related to the previously described modulation of the OPG/RANKL balance, inhibition of osteoclast activity, and promotion of osteoblast function.

Clinical research also shows a positive trend. Randomized controlled trials indicate that supplementation with EPA and DHA can significantly reduce serum bone resorption markers (e.g., CTX-1) and moderately increase bone formation markers (e.g., P1NP) in postmenopausal women, suggesting the potential to bidirectionally regulate bone metabolism, tilting the balance toward bone formation (39). Regarding their direct impact on BMD, results across studies are not entirely consistent. A meta-analysis incorporating 10 RCTs noted a slight positive trend toward improving lumbar spine BMD, particularly with longer intervention durations (>12 months) (40). This suggests the effect, if present, is likely modest and may be more pronounced in populations with low baseline ω-3 levels. The association with reduced hip fracture risk from large observational studies (41) is promising, but requires confirmation in large-scale RCTs with fracture as the primary endpoint.

ω-3 PUFAs also show application potential in specific types of osteoporosis. For instance, in animal models of glucocorticoid-induced osteoporosis (GIOP), DHA was found to partially antagonize glucocorticoid-induced inhibition of bone formation and destruction of bone structure (42). In disuse or unloading-induced bone loss models, ω-3 PUFA supplementation has also been confirmed to attenuate the exacerbated bone resorption associated with lack of mechanical loading, likely via its anti-inflammatory properties (43). Furthermore, considering that aging itself is accompanied by chronic inflammation (“inflammaging”), a key driver of bone loss, a more effective approach to complex issues like osteosarcopenia in the elderly may involve combined interventions rather than single-nutrient supplements. Combining ω-3 PUFAs, adequate high-quality protein (particularly leucine-rich), and vitamin D constitutes a multi-targeted “nutritional combination.” Existing research suggests that for the elderly or individuals with sarcopenia, such a combined supplementation regimen may be more effective in improving muscle mass, strength, and physical function than any single component (44). The underlying mechanisms are complementary: ω-3 PUFAs optimize the overall anabolic signaling environment, protein provides the building blocks and strongly activates the mTORC1 pathway, while vitamin D directly regulates muscle and bone cell function through its receptor and promotes calcium absorption and utilization. The synergy among the three acts on different dimensions of the bone-muscle metabolic axis.

### 4.2 Symptom relief and structural protection in osteoarthritis

Evidence for the application of ω-3 PUFAs in osteoarthritis (OA) management primarily centers on symptom improvement, with promising indications for structural protection. Their mechanisms for pain relief are multifaceted: beyond indirectly reducing joint inflammation through systemic anti-inflammatory effects, their metabolites (e.g., resolvin E1) can directly act on peripheral sensory neurons, inhibiting the release of pain mediators like substance P and CGRP, producing a direct peripheral analgesic effect (45). Clinical trials show that daily supplementation with at least 1 gram of EPA + DHA for 3–6 months can significantly reduce joint pain and morning stiffness in patients with knee OA, improving subjective scores such as WOMAC and VAS (9).

Of greater interest is the potential of ω-3 PUFAs for structural protection beyond mere analgesia. Animal OA models confirm that ω-3 PUFA supplementation can reduce cartilage damage, synovial inflammation, and osteophyte formation (46). In humans, although long-term (e.g., several years) data on radiographic structural improvement are still limited, observational studies have found an association between higher dietary ω-3 intake and a slower rate of joint space narrowing (47). Furthermore, interventional studies have observed a decrease in serum or urinary levels of biomarkers reflecting cartilage degradation (e.g., CTX-II and COMP) following ω-3 PUFA supplementation (9), providing biochemical evidence suggesting a potential to slow cartilage matrix destruction.

Given their favorable safety profile, ω-3 PUFAs hold a place in long-term pharmaceutical management strategies for OA. Evidence suggests that regular intake may reduce patients’ reliance on or required dosage of non-steroidal anti-inflammatory drugs (NSAIDs) (48). For patients unable to use NSAIDs long-term due to gastrointestinal or cardiovascular risks, ω-3 PUFAs can serve as a beneficial adjunct or partial alternative. Combined with other analgesics like acetaminophen, they may contribute to achieving better pain control and functional improvement.

### 4.3 Adjunctive role in perioperative recovery and trauma repair

The immunomodulatory and metabolic regulatory functions of ω-3 PUFAs hold significant application value in the complex process of recovery from orthopedic surgery and trauma. The stress response following major surgery often leads to systemic inflammation and a hypercatabolic state, triggering insulin resistance and skeletal muscle atrophy. Providing “immunonutrition” support rich in ω-3 PUFAs during the perioperative period (typically initiated preoperatively) has been shown to help mitigate the inflammatory response, improve insulin sensitivity, and thereby reduce postoperative muscle loss. This is particularly meaningful for elderly patients undergoing major surgeries like hip or knee arthroplasty (49).

In surgeries requiring internal fixation or bone grafting, achieving stable integration between the implant and host bone (osseointegration) is crucial for success. Animal studies provide new insights: both local application and systemic supplementation of ω-3 PUFAs have been observed to promote new bone formation around implants, increasing the bone-implant contact rate and mechanical bonding strength. The mechanisms are related to the pro-repair microenvironment they foster (via SPMs, etc.) and the direct promotion of osteoblast activity (50).

Furthermore, ω-3 PUFAs may play an auxiliary role in challenging infectious bone diseases like chronic osteomyelitis. While they cannot replace antibiotics, their metabolite SPMs can enhance the phagocytic and bactericidal capacity of macrophages while actively promoting inflammation resolution (51). This property of “immunomodulation” rather than “immunosuppression” may offer a promising nutritional adjunctive strategy for controlling infection, mitigating accompanying inflammatory bone destruction, and promoting healing, adding a new dimension to comprehensive treatment plans.

## 5 Exercise performance and muscle health

### 5.1 Promotion of muscle protein metabolism and adaptation

Muscle growth and repair fundamentally depend on the balance between protein synthesis and breakdown. In this process, ω-3 PUFAs act more as a “metabolic modulator” or “signal amplifier.” Clinical research provides direct evidence: a randomized controlled trial found that healthy middle-aged and older adults supplementing with approximately 3.4 grams of EPA + DHA daily for 8 weeks experienced about a 30% increase in the skeletal muscle anabolic response to amino acids and insulin (19). Behind this “sensitizing” effect lies the ability of ω-3 PUFAs to optimize the cell membrane environment and enhance insulin/IGF-1 receptor binding affinity, promoting receptor autophosphorylation. This initiates downstream signaling, enabling the protein synthesis-regulating mTORC1 pathway to be activated more efficiently.

Interestingly, ω-3 PUFAs may also exhibit synergistic effects with other nutrients. For example, their interaction with the essential amino acid leucine (a powerful direct activator of mTORC1) is theoretically promising. Combining the improved overall anabolic “background” provided by ω-3 PUFAs with the “trigger” signal from leucine may produce additive effects in combating age-related or disuse muscle atrophy (19). This hypothesized synergy is based on complementary mechanisms—ω-3 PUFAs improving the anabolic ‘background’ sensitivity and leucine providing a strong direct ‘trigger’ for mTORC1. While theoretically promising and supported by some pre-clinical data, this additive effect requires direct confirmation in well-designed human intervention trials, particularly in populations where anabolic resistance is prevalent, such as the elderly.

Beyond regulating protein synthesis, ω-3 PUFAs may also be involved in the muscle repair process post-injury. Animal studies indicate that ω-3 PUFA supplementation (especially DHA) can improve muscle regeneration after injury (52). The mechanisms likely involve two aspects: first, through their anti-inflammatory properties, reducing the local inflammatory environment at the injury site, removing obstacles to the activation and proliferation of satellite cells (muscle stem cells); second, potentially directly or indirectly influencing signaling pathways like Notch and Wnt that regulate satellite cell fate. This suggests that ω-3 PUFAs not only aid in “muscle building” but may also promote functional recovery of muscle after intense training or acute injury. It is also worth noting that the ergogenic effects of ω-3 supplementation on muscle mass and strength in young, healthy, resistance-trained individuals are less consistent than in older or untrained populations, suggesting that the benefits may be most pronounced in states of existing anabolic resistance or heightened inflammation.

### 5.2 Optimization of energy metabolism and fatigue management in endurance exercise

For endurance sports, the efficiency of energy supply is paramount. The role of ω-3 PUFAs in this area focuses primarily on enhancing fat oxidation capacity and improving metabolic flexibility. The mechanism involves the regulation of skeletal muscle mitochondria: supplementation with EPA and DHA can upregulate the expression of PGC-1*α* (a key regulator of mitochondrial biogenesis), promoting the generation of new, better-functioning mitochondria (53). Simultaneously, as ligands for peroxisome proliferator-activated receptor alpha (PPAR-α), they can also enhance the activity of enzymes related to fatty acid transport and oxidation (54). These changes collectively enable athletes to utilize fat more efficiently for energy during exercise, helping to conserve valuable muscle glycogen stores and delay fatigue associated with glycogen depletion, commonly known as “hitting the wall.” While these metabolic shifts (increased fat oxidation) are favorable, translating them directly into improved endurance performance, such as faster race times, requires further confirmation in well-controlled athletic populations, as the existing evidence is mixed.

Additionally, exercise itself is a potent physiological stressor, often accompanied by acute inflammation and oxidative damage, which are sources of fatigue and affect recovery speed. The classic anti-inflammatory and antioxidant effects of ω-3 PUFAs are applicable here. Supplementation with ω-3 PUFAs has been confirmed to lower post-exercise circulating levels of pro-inflammatory cytokines (e.g., IL-6) and muscle damage markers (e.g., creatine kinase, CK), aiding in reducing exercise-induced muscle micro-damage and inflammatory responses and accelerating the recovery of physical function (55).

Another exploratory direction is the potential impact of ω-3 PUFAs on central fatigue. Central fatigue is associated with altered balances of neurotransmitters in the brain, such as serotonin. DHA, as a core structural lipid in brain and neuronal membranes, may influence fatigue perception and exercise motivation by maintaining blood–brain barrier integrity (reducing interference from peripheral inflammatory factors entering the brain) and regulating neurotransmitter system function (56). Although direct human performance evidence for this mechanism is still accumulating, it provides a plausible theoretical perspective for explaining the potential benefits of ω-3 PUFAs in extreme or prolonged endurance events.

### 5.3 Potential role in sports injury prevention and recovery

The health of the movement system depends not only on muscles but also on connective tissues like tendons and ligaments. Preliminary studies suggest that ω-3 PUFAs may help maintain connective tissue health by influencing collagen metabolism, reducing inflammation-induced inhibition of collagen synthesis through their anti-inflammatory action, and potentially optimizing collagen fiber cross-linking quality by modulating lysyl oxidase activity (57). This implies that long-term adequate ω-3 PUFA intake may help improve the mechanical properties of connective tissues, offering some nutritional support for preventing overuse injuries like tendinopathy.

In the more common post-exercise response—delayed onset muscle soreness (DOMS)—ω-3 PUFAs also show alleviating potential. Studies confirm that supplementing with ω-3 PUFAs (typically >2 grams daily) before and after strenuous eccentric exercise has been shown to reduce subjective pain perception and biomarkers of muscle damage (58). However, this reduction in soreness does not consistently lead to measurable improvements in subsequent muscle function or performance.

Particularly noteworthy is their protective prospects in neurological sports injuries like concussion. A substantial body of preclinical research provides a strong rationale for the neuroprotective potential of DHA in concussion. Following sports-related concussion, secondary neuroinflammation is a core mechanism leading to persistent symptoms and long-term sequelae. Preclinical (animal) studies provide strong evidence: supplementation with high doses of DHA before and after injury can significantly reduce neuroinflammation, decrease neuronal death, and improve subsequent cognitive and motor function in model animals (59). DHA is not only a direct precursor for neuroprotective pro-resolving mediators but is also essential for maintaining neuronal membrane stability and function. These findings lay a solid scientific foundation for future clinical trials, which are urgently needed to determine its efficacy as a preventive or therapeutic agent in human athletes.

## 6 Exploration of special application scenarios

### 6.1 As a metabolic regulator of the “bone-muscle axis” and a combined intervention strategy

Bone and muscle are not merely anatomical neighbors; they form a functionally integrated “metabolic community” through complex endocrine and paracrine signaling networks, a concept now widely recognized as the bone-muscle axis (60). This bidirectional crosstalk is mediated by multiple factors. For instance, myokines released by contracting muscle, such as irisin, interleukin-6 (IL-6), and insulin-like growth factor-1 (IGF-1), can directly influence bone metabolism by modulating osteoblast and osteoclast activity (61). Conversely, osteokines secreted from bone, including osteocalcin, sclerostin, and fibroblast growth factor 23 (FGF-23), feedback to regulate muscle mass, function, and energy metabolism (62). This intimate coupling ensures that under physiological conditions, the development, maintenance, and adaptation of bone and muscle are coordinately regulated to meet mechanical and metabolic demands.

However, this tightly regulated axis is highly susceptible to disruption by pathological stimuli. Chronic low-grade inflammation emerges as a key “disruptor” that simultaneously erodes the health of both bone and muscle. Elevated levels of pro-inflammatory cytokines, particularly tumor necrosis factor-alpha (TNF-*α*) and IL-6, have been shown to directly impair this bidirectional crosstalk (63). On the bone side, these cytokines activate NF-κB signaling in osteoclast precursors, promoting osteoclastogenesis and bone resorption, while simultaneously suppressing osteoblast differentiation and bone formation (64). On the muscle side, TNF-α and IL-6 activate proteolytic pathways such as the ubiquitin-proteasome system via NF-κB, leading to muscle protein breakdown and reduced muscle mass, while also inducing insulin resistance that impairs the anabolic response to nutrients (65). Thus, inflammation creates a vicious cycle where deterioration in one tissue exacerbates decline in the other.

In this context, the therapeutic value of ω-3 PUFAs lies precisely in their ability to target this common inflammatory denominator. Through the multiple mechanisms detailed in Section 3—including inhibition of NF-κB and NLRP3 inflammasome activation, and the generation of specialized pro-resolving mediators—ω-3 PUFAs exert potent systemic anti-inflammatory and pro-resolving effects that positively impact the microenvironment of both organs simultaneously (14, 16). By reducing circulating levels of TNF-*α* and IL-6, ω-3 PUFAs not only inhibit osteoclast-driven bone resorption but also improve muscle insulin sensitivity and protein anabolic metabolism (19, 39). This dual action is not merely additive but potentially synergistic: preserving muscle mass helps maintain mechanical loading on bone, while maintaining bone health supports the structural integrity necessary for muscle function.

Therefore, ω-3 PUFA supplementation should be conceptualized as a strategy for synergistically maintaining the overall health of the “bone-muscle unit” at the metabolic level. This framework has direct translational implications for several specific clinical contexts where the bone-muscle axis is compromised:

Osteosarcopenia: This condition, characterized by the concurrent presence of osteoporosis and sarcopenia in aging individuals, exemplifies the clinical relevance of the bone-muscle axis (66). The shared inflammatory pathogenesis makes it an ideal target for ω-3 PUFA intervention. By simultaneously addressing the inflammatory drivers of both bone loss and muscle wasting, ω-3 PUFAs offer a unified therapeutic approach that targets the common root cause rather than treating each condition in isolation.

Disuse Atrophy: In conditions of unloading such as prolonged bed rest, limb immobilization, or spaceflight, the mechanical coupling within the bone-muscle axis is disrupted, leading to accelerated bone resorption and muscle protein breakdown (43). Inflammation associated with unloading further exacerbates this catabolic state. ω-3 PUFA supplementation has been shown in simulated microgravity studies to mitigate both bone loss and muscle atrophy, likely by counteracting the inflammation that amplifies unloading-induced damage (43).

RED-S (Relative Energy Deficiency in Sport): In athletes, particularly females, low energy availability disrupts the endocrine signals within the bone-muscle axis, suppressing bone formation and muscle protein synthesis (67). ω-3 PUFAs offer a dual benefit in this context: as a high-energy-density nutrient, they can help meet energy requirements, while their anti-resorptive properties provide partial skeletal protection against the detrimental effects of energy deficit (68).

Aging: The progressive low-grade inflammation characteristic of aging, termed “inflammaging,” is a major driver of age-related decline in both bone and muscle (69). By combating inflammaging, ω-3 PUFAs help preserve the functional capacity of both tissues, supporting mobility, strength, and quality of life in the elderly population.

**Figure 3:**
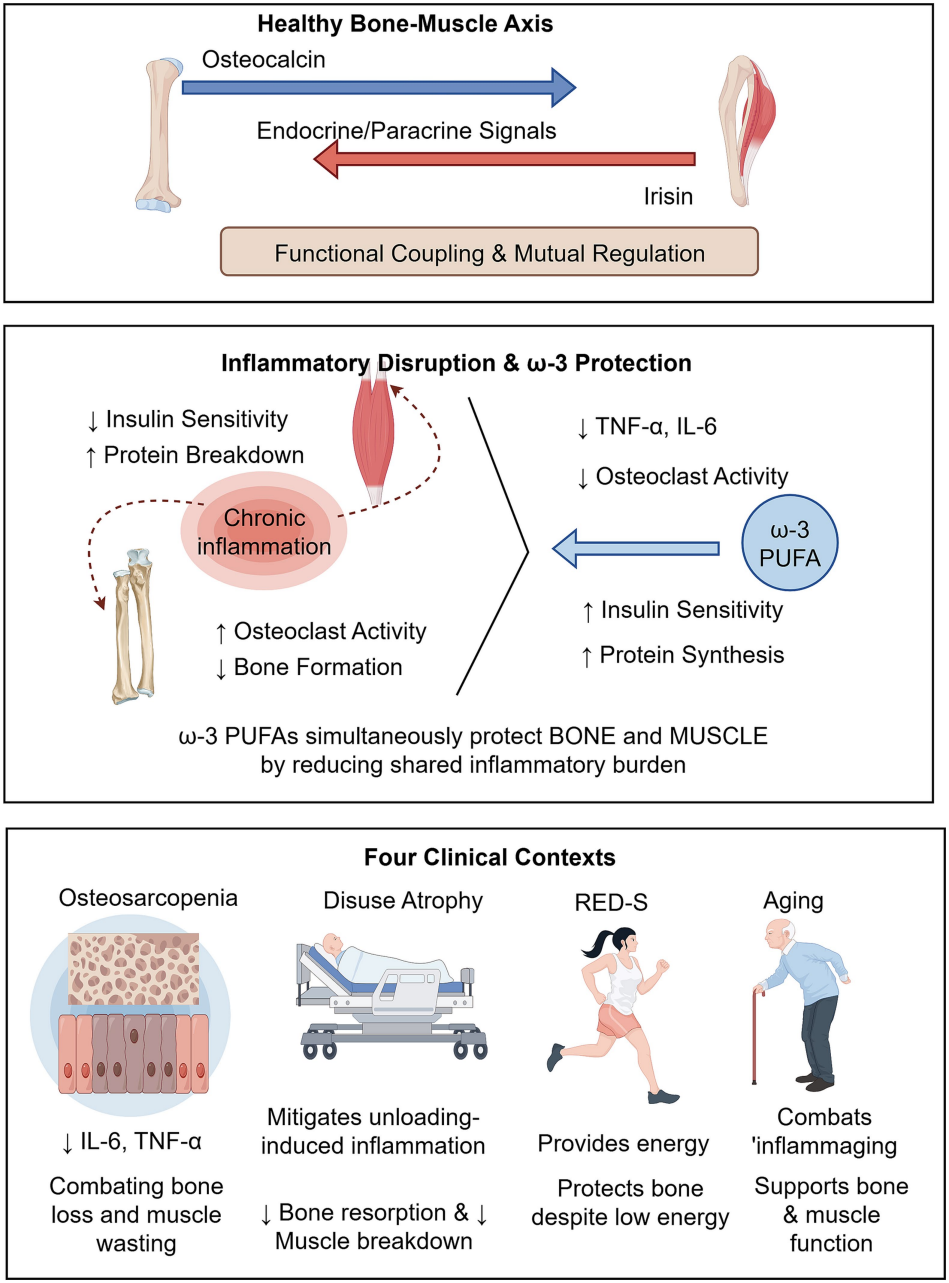
The bone-muscle axis and the therapeutic role of ω-3 PUFAs in clinical contexts. This figure illustrates the concept of the bone-muscle axis and elucidates how ω-3 polyunsaturated fatty acids (PUFAs) exert systemic therapeutic effects that simultaneously benefit both bone and muscle tissues across four clinically relevant scenarios. The figure is organized into three horizontal sections. Section 1 (Top): Healthy bone-muscle axis. Depicts the physiological bidirectional crosstalk between bone and muscle, mediated by myokines (e.g., irisin, IL-6, IGF-1) released from muscle and osteokines (e.g., osteocalcin, sclerostin, FGF-23) secreted from bone. This intimate coupling ensures coordinated regulation of bone and muscle under physiological conditions. Section 2 (Middle): Inflammatory disruption and ω-3 protection. Left side illustrates chronic low-grade inflammation (elevated TNF-*α*, IL-6) as a key disruptor that simultaneously damages both tissues: promoting osteoclastogenesis and bone resorption while inducing muscle protein breakdown and insulin resistance. Right side shows ω-3 PUFAs exerting systemic anti-inflammatory effects by reducing TNF-α and IL-6, thereby inhibiting bone resorption and improving muscle insulin sensitivity and protein synthesis. The blue arrows represent this protective action, intercepting and blocking inflammatory signals. Section 3 (Bottom): Therapeutic applications in four clinical contexts. Translates these mechanisms into specific conditions where the bone-muscle axis is compromised: Osteosarcopenia: ω-3 PUFAs simultaneously counter inflammatory drivers of age-related bone loss and muscle wasting. Disuse atrophy: ω-3 PUFAs mitigate unloading-induced inflammation, reducing accelerated bone resorption and muscle protein breakdown. RED-S (Relative energy deficiency in sport): ω-3 PUFAs provide high-energy-density nutrition with anti-resorptive properties, offering skeletal protection against energy deficit. Aging: ω-3 PUFAs combat “inflammaging,” preserving functional capacity of both bone and muscle. Together, this figure provides a unified framework for understanding how ω-3 PUFAs, through systemic anti-inflammatory effects, simultaneously protect bone and muscle health across a spectrum of pathological and physiological states. RED-S, relative energy deficiency in sport; TNF-α, tumor necrosis factor-alpha; IL-6, interleukin-6; IGF-1, insulin-like growth factor-1; FGF-23, fibroblast growth factor 23. (This figure was created using www.figdraw.com. ID: WUOOScffcf).

In summary, conceptualizing ω-3 PUFA action through the lens of the bone-muscle axis provides a unified framework for understanding their pleiotropic benefits and guides the development of integrated nutritional strategies for conditions that simultaneously affect bone and muscle health. Thereby supporting the functional capacity of both tissues. (This concept is illustrated in Figure 3).

### 6.2 Precision nutritional support in athletic populations

#### 6.2.1 Potential role in the female athlete triad

The Female Athlete Triad (Relative Energy Deficiency in Sport, RED-S) is characterized by low energy availability, menstrual dysfunction, and low bone mineral density, posing a serious threat to athlete health (68). Low energy availability is the core driver, directly accelerating bone turnover and bone loss. In this scenario, ω-3 PUFAs may play a dual supportive role. First, as a nutrient with high energy density, they can help maintain necessary total energy intake during strict weight control periods. Second, and more importantly, their inherent anti-resorptive and anti-inflammatory properties may provide an additional layer of nutritional protection for bone health, potentially buffering the negative impact of low energy status on the skeleton.

#### 6.2.2 Addressing overtraining syndrome

Overtraining syndrome is marked by persistent fatigue, decreased performance, and impaired recovery capacity, underpinned by long-term systemic inflammation, oxidative stress, and neuroendocrine dysregulation (70). ω-3 PUFAs, particularly their derived specialized pro-resolving mediators (SPMs), can actively guide the physiological resolution of inflammation (14). Furthermore, their stabilizing effect on neuronal cell membranes and potential modulation of neurotransmitter systems may help improve the mood state and neural fatigue often associated with overtraining (56). Therefore, regular supplementation with ω-3 PUFAs may enhance the body’s anti-inflammatory and antioxidant reserves during prevention phases and potentially accelerate the normalization of physiological parameters during recovery.

### 6.3 Application potential in extreme environment medicine

In high-stress extreme environments such as spaceflight (microgravity), prolonged bed rest, or military operations, the skeletal muscle system faces rapid disuse atrophy and metabolic dysregulation, a core challenge in aerospace and military medicine. Supplementation with ω-3 PUFAs (daily doses typically in the range of 2–4 grams EPA + DHA) has been preliminarily proven to be a promising nutritional countermeasure. For example, a 6-week simulated microgravity (head-down bed rest) study showed that participants in the supplementation group experienced about 50% less loss in tibial bone mineral density and significantly less muscle atrophy compared to the control group (43). The protective mechanisms are believed to be related to the inhibition of NF-κB-mediated inflammatory bone resorption, reduction in muscle protein catabolism, and improvement of insulin resistance.

These extreme environments are often accompanied by intense physiological and psychological stress, leading to elevated levels of stress hormones like cortisol, which in turn drive excessive, destructive inflammatory responses. The mechanism of action of ω-3 PUFAs and their SPMs (e.g., resolvin D series) here is distinctive: they do not simply “suppress” the immune response but provide an endogenous, programmed pathway for inflammation regulation and recovery promotion (14). This mode of action helps maintain more stable immune homeostasis under prolonged stress, mitigating the cumulative damage of chronic inflammation to physiological function, thereby potentially enhancing an individual’s operational endurance and overall health resilience in extreme environments. Table 3 summarizes the applications and development potential of ω-3 PUFAs in orthopedics and sports medicine.

**Table 3:** Potential applications of ω-3 PUFAs in orthopedics and sports medicine.

Stage level	Core findings	Translational directions	References
Basic research	1. Membrane remodeling and mechanobiology: Optimize mechanical signal sensing.	Enhance the adaptability of the musculoskeletal system during exercise and rehabilitation training through nutritional approaches.	(12, 13, 30)
	2. Lipid mediator profile reshaping: Competitively replace arachidonic acid (AA), reduce prostaglandin E₂ (PGE₂) production, and generate specialized pro-resolving mediators (SPMs): Guide inflammation resolution and tissue repair.	Explain its mechanism of action in chronic inflammatory diseases and recovery after acute injury.	(14, 32, 33)
	3. Direct inhibition of pro-inflammatory pathways: Suppress NF-κB pathway and NLRP3 inflammasome activation, reduce the production of pro-inflammatory cytokines such as tumor necrosis factor-α (TNF-α), interleukin-6 (IL-6), and interleukin-1β (IL-1β).	Provide pathways for regulating inflammaging through nutritional intervention.	(15, 16)
	4. Targeted regulation of cellular functions: Activate the Wnt/β-catenin pathway to promote osteogenic differentiation and bone. Formation RANKL/OPG balance to inhibit bone resorption. enhance the PI3K/Akt/mTORC1 signal to promote muscle protein synthesis balance matrix metalloproteinases (MMPs)/tissue inhibitors of metalloproteinases (TIMPs) to maintain cartilage homeostasis.	Offer cytological evidence for the prevention and treatment of osteoporosis, sarcopenia and osteoarthritis.	(17–20, 37)
Preclinical and translational research	1. Disease model validation: Confirmed to increase bone mineral density (BMD) and improve bone biomechanics in ovariectomized (OVX) rat models; showed reduction in cartilage damage and inflammation in osteoarthritis models.	Provide preliminary evidence for human clinical trials targeting diseases such as osteoporosis and osteoarthritis.	(38, 46)
	2. Exploration of special models: Demonstrated potential in models of glucocorticoid-induced osteoporosis (GIOP), disuse-induced bone loss,muscle injury regeneration, concussion neuroprotection, and implant osseointegration.	Expand application research in scenarios including GIOP, sports injuries, neuroprotection, and orthopedic implants.	(42, 43, 50, 52, 59)
	3. Exploration of novel delivery systems: Phospholipid-bound ω-3 (e.g., lysophosphatidylcholine-DHA, LPC-DHA) may achieve higher tissue targeting through the MFSD2A transporter; ω-3-loaded biomaterial coatings can promote osseointegration.	Guide the development of highly targeted supplements and bioactive implants.	(50, 66, 67)
Clinical research and application	1. Orthopedic disease prevention and treatment: Supplementation can improve bone turnover markers; long-term supplementation may show a positive trend in lumbar spine BMD; observational studies suggest an association with lower hip fracture risk. Daily intake of ≥1 g EPA+DHA can significantly alleviate osteoarthritis pain, improve function, and may reduce cartilage degradation biomarkers. As a component of immunonutrition, it helps reduce postoperative inflammation and muscle loss.	Serve as an auxiliary nutritional strategy for symptom management of orthopedic diseases.	(9, 39–41, 49)
	2. Sports health: Enhances skeletal muscle protein synthetic response in middle-aged and elderly adults; alleviates post-exercise muscle soreness and inflammation. May optimize energy metabolism by promoting mitochondrial biogenesis and fat oxidation. Shows neuroprotective potential for concussion (prominent preclinical evidence).	Used to optimize muscle protein anabolism and accelerate post-exercise recovery; may also serve as a potential nutritional adjuvant in concussion management	(19, 53, 54, 58, 59)
	3. Special scenarios: Combined supplementation with protein and vitamin D may be superior to single-component supplementation in improving musculoskeletal function in the elderly; demonstrated application potential in female athletes with energy restriction and simulated microgravity environments.	Act as a core component of multimodal nutritional interventions. Consider inclusion in comprehensive nutritional management plans for relative energy deficiency in sport (RED-S) or overtraining syndrome. Candidate nutritional countermeasure against disuse atrophy	(44, 45, 63)
Future translational challenges and direction	1. Protocol optimization: Optimal dosage, EPA: DHA ratio, intervention timing, and supplement form (e.g., phospholipid-bound type) remain to be clarified.	Conduct RCTs targeting different health goals to determine optimal protocols.	(70)
	2. Individualized strategies: Individual responses are influenced by genetic factors (FADS gene polymorphisms), gut microbiota, baseline nutritional status, and other factors.	Integrate multi-omics data to develop precision nutrition strategies.	(68, 69)
	3. Interdisciplinary integration: Synergistic effects of combined interventions with physical therapy (e.g., vibration training), biomaterials, and drugs need to be explored.	Carry out clinical studies on nutrition-physical combined interventions; develop innovative products such as bioactive implants.	(71)

This table systematically summarizes the multi-dimensional application potential of ω-3 polyunsaturated fatty acids in improving bone metabolism, alleviating osteoarthritis symptoms, enhancing muscle synthesis and recovery, and addressing special physiological and environmental challenges, while also highlighting future translational challenges in personalized strategies and interdisciplinary integration.

## 7 Controversies, limitations, and future directions

### 7.1 Optimization of dosage, form, and delivery routes

Although the benefits of ω-3 PUFAs are widely recognized, uncertainties remain regarding the optimal protocol for clinical application. Current research predominantly employs long-term supplementation strategies (weeks to months) aimed at steadily elevating tissue ω-3 levels, which is undoubtedly a robust foundational approach. However, in specific scenarios such as acute injury (e.g., muscle strain, early fracture) or the perioperative period, a more pressing question arises: does a critical “acute intervention window” exist? During this period, could short-term, higher-dose supplementation regimens more rapidly modulate local inflammation and thereby optimize the repair process? Future research needs to carefully compare the actual impact of different intervention timings (e.g., pre-injury preventive supplementation, immediate or delayed post-injury supplementation) on tissue repair quality and speed.

Regarding supplement formulations, traditional fish oils (triglyceride or ethyl ester forms) are facing new challenges and opportunities. Emerging evidence suggests that phospholipid-form ω-3 (e.g., DHA from krill oil or certain algal sources) may offer higher bioavailability, particularly for tissues requiring passage across special biological barriers. A key mechanism is that DHA in the lysophosphatidylcholine (LPC) form can be transported more efficiently across the blood–brain barrier via the specific MFSD2A transporter, which is crucial for neuroprotection (71). Interestingly, MFSD2A expression is not limited to brain vascular endothelium; it is also found in capillaries of various tissues, including bone marrow (72). This raises an intriguing hypothesis: bone tissue microvascular endothelium may similarly possess the capability for efficient LPC-DHA uptake, thus providing a potential molecular portal for the “bone-targeted” delivery of ω-3. Investigating the permeability of the so-called “blood-bone barrier” to phospholipid-form ω-3 and its targeting efficiency to bone is a promising frontier direction worthy of in-depth exploration.

### 7.2 Individual variability and moving towards precision nutrition

Significant individual variability in response to ω-3 supplementation is a major obstacle to maximizing its clinical benefits. The key to overcoming this bottleneck lies in developing precision nutrition strategies.

First, genetic background plays a fundamental role. Common single nucleotide polymorphisms (SNPs) in the FADS1/FADS2 genes, which encode the *Δ*-5 and Δ-6 desaturase enzymes, significantly affect an individual’s efficiency in converting plant-derived ALA to long-chain EPA and DHA (73). Individuals carrying alleles associated with low conversion efficiency may rely more on direct dietary intake of EPA and DHA rather than ALA supplementation. Therefore, future clinical trials should incorporate FADS genotyping to elucidate optimal supplement forms and dosages for different genetic phenotypes.

Second, the gut microbiota represents another critical layer of individual variability. A bidirectional, dynamic interaction exists between the gut microbiota and dietary ω-3 PUFAs. Dietary supplementation with ω-3 PUFAs (especially EPA and DHA) can increase the abundance of beneficial bacteria (e.g., Lactobacillus, Bifidobacterium, Akkermansia) while reducing inflammation-associated flora. The gut bacteria themselves possess desaturase and elongase activities, allowing partial conversion of plant-derived ALA to long-chain PUFAs (e.g., EPA, DPA), although the contribution of this pathway to the human host’s overall status is likely limited. Moreover, the microbiota can transform ω-3 PUFAs into oxidized lipid mediators with local or systemic activity. They can also indirectly regulate the host’s absorption efficiency and tissue distribution of ω-3 PUFAs by influencing bile acid metabolism, gut inflammatory status, and intestinal epithelial integrity (74). This raises the possibility that targeted modulation of the gut microbiota via strategies like probiotics/prebiotics might optimize host utilization efficiency of ω-3 PUFAs. Integrating genomic and microbiome information will be central to constructing individualized nutrition plans in the future.

To operationalize this, we propose a framework where an individual’s baseline ω-3 index, FADS1 genotype, and gut microbiota profile could guide supplementation. For example:

Step 1: Assess Baseline ω-3 Index. An index <4% suggests a need for supplementation, while 4–8% might indicate maintenance.
Step 2: FADS1 Genotyping. Individuals with the “low-conversion” (non-optimizing) haplotype for the FADS1/2 gene cluster would be strongly advised to take preformed EPA/DHA rather than ALA, and might require higher doses to achieve a target ω-3 index.
Step 3: Define Goal and Choose Formula. For anti-inflammatory goals (e.g., in OA), a higher EPA: DHA ratio might be prioritized. For neuroprotection or cell membrane integrity (e.g., concussion recovery), a DHA-rich phospholipid formulation could be preferred.
Step 4: Monitor and Adjust. After 3–6 months of supplementation, the ω-3 index is re-measured. If the target is not met, the dose or formulation (e.g., switching to a phospholipid form for better bioavailability) could be adjusted. Future trial designs, such as a “basket trial” assigning different ω-3 formulations based on FADS genotype or baseline inflammatory markers, could test this framework’s efficacy in specific outcomes like muscle protein synthesis or fracture healing.

This framework transforms the descriptive concept into a testable clinical strategy.

### 7.3 Prospects for interdisciplinary translational research

To fully realize the potential of ω-3 PUFAs, research needs to move beyond traditional oral supplementation paradigms and embrace innovative interdisciplinary translational approaches.

In the field of biomaterials, loading ω-3 PUFAs or their active derivatives onto the surface of orthopedic implants (e.g., titanium alloys) using nanotechnology to create functional coatings is a highly promising direction. This design enables local, sustained release at the implantation site, concurrently exerting multiple effects: promoting osseointegration, providing anti-inflammatory action, and potentially modulating the local immune microenvironment. Animal research has demonstrated its potential (50), but its safety, long-term efficacy, and carrier degradation behavior require further in-depth study.

In nutritional product development, current commercial products have varying EPA: DHA ratios, but the two seem to have somewhat distinct physiological emphases. Existing evidence tends to suggest that EPA plays a more prominent role in modulating systemic inflammation and muscle metabolism, while DHA is crucial for neuroprotection and cell membrane function (75). Therefore, a core unanswered question is: do optimal EPA: DHA ratios exist for different health goals (e.g., anti-inflammation, muscle building, neuroprotection)? This requires answers from well-designed head-to-head comparative studies.

Furthermore, combining nutritional interventions with physical modalities may yield synergistic effects. For example, exploring whether combining ω-3 PUFA supplementation with whole-body vibration training or blood flow restriction training produces a “1 + 1 > 2” effect in improving muscle strength, bone density, and functional recovery in the elderly or postoperative rehabilitation populations. The theoretical basis is that ω-3 PUFAs may enhance cellular sensitivity to mechanical stimuli and subsequent anabolic responses by optimizing cell membrane properties and signal transduction (76).

## 8 Conclusion

In summary, based on existing evidence, ω-3 PUFAs have established their key nutritional role in maintaining skeletal muscle system health. Their actions extend far beyond traditional understanding, profoundly influencing the homeostasis, adaptation, and repair of tissues like bone, muscle, and cartilage through a multi-dimensional network ranging from cell membrane modification and lipid mediator reprogramming to intracellular signal regulation.

At the application level, adequate ω-3 intake demonstrates clear value in preventing and managing osteoporosis and osteoarthritis, as well as in optimizing exercise performance and accelerating injury recovery. Their potential in special scenarios such as age-related sarcopenia, the Female Athlete Triad, and spaceflight further broadens their applicability.

However, the translation of their clinical potential truly faces the core challenge of “individual variability.” Future research must strive to bridge this gap by integrating dynamic biomarkers such as FADS genotype, gut microbiota features, and the erythrocyte membrane ω-3 index to construct an individualized nutritional decision-making framework. Simultaneously, the development of innovative delivery systems (e.g., phospholipid formulations, local delivery agents, biomaterial coatings) and multimodal combination strategies with other therapeutic modalities (e.g., specific exercise regimens) represent the most promising breakthrough directions in this field.

In conclusion, the evidence positions ω-3 PUFAs as significant modulators of musculoskeletal health, operating through interconnected pathways that influence nutrition, metabolism, immunity, and mechanobiology. This understanding elevates their role from a simple dietary supplement to a key nutritional factor that can be strategically employed in clinical and sports settings.
